# Mendelian Randomization Analysis With Multiple Genetic Variants Using Summarized Data

**DOI:** 10.1002/gepi.21758

**Published:** 2013-09-20

**Authors:** Stephen Burgess, Adam Butterworth, Simon G Thompson

**Affiliations:** Department of Public Health and Primary Care, University of CambridgeCambridge, United Kingdom

**Keywords:** Mendelian randomization, instrumental variables, genome-wide association study, causal inference, weak instruments

## Abstract

Genome-wide association studies, which typically report regression coefficients summarizing the associations of many genetic variants with various traits, are potentially a powerful source of data for Mendelian randomization investigations. We demonstrate how such coefficients from multiple variants can be combined in a Mendelian randomization analysis to estimate the causal effect of a risk factor on an outcome. The bias and efficiency of estimates based on summarized data are compared to those based on individual-level data in simulation studies. We investigate the impact of gene–gene interactions, linkage disequilibrium, and ‘weak instruments’ on these estimates. Both an inverse-variance weighted average of variant-specific associations and a likelihood-based approach for summarized data give similar estimates and precision to the two-stage least squares method for individual-level data, even when there are gene–gene interactions. However, these summarized data methods overstate precision when variants are in linkage disequilibrium. If the *P*-value in a linear regression of the risk factor for each variant is less than 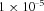, then weak instrument bias will be small. We use these methods to estimate the causal association of low-density lipoprotein cholesterol (LDL-C) on coronary artery disease using published data on five genetic variants. A 30% reduction in LDL-C is estimated to reduce coronary artery disease risk by 67% (95% CI: 54% to 76%). We conclude that Mendelian randomization investigations using summarized data from uncorrelated variants are similarly efficient to those using individual-level data, although the necessary assumptions cannot be so fully assessed.

## Introduction

Mendelian randomization is a technique for using genetic variants to estimate the causal effect of a modifiable risk factor from observational data [Davey Smith and Ebrahim, 2003]. It has recently been used to strengthen the evidence for causal roles in coronary heart disease of interleukin-6 [Swerdlow et al., 2012] and lipoprotein(a) [Kamstrup et al., 2009]. A limitation of Mendelian randomization is that genetic variants often only explain a small fraction of the variation in the risk factor of interest [Davey Smith and Ebrahim, 2004], so that assessing some causal associations requires sample sizes running into tens of thousands to obtain adequate power [Schatzkin et al., 2009]. This problem can be partially redressed by the use of multiple genetic variants [Palmer et al., 2011]. If each variant explains additional variation in the risk factor, then a combined causal estimate using all of the variants will have greater precision than the estimate from any of the individual variants [Pierce et al., 2011].

One potential source of such data is genome-wide association (GWA) studies, which examine the associations of many genetic variants with a trait. Many large GWA study consortia have been assembled, with sample sizes in some cases running into hundreds of thousands [Ehret et al., 2011]. Individual-level data on study participants are not always available due to issues of practicality and confidentiality of data-sharing on such a large scale. Presentations of results from GWA studies often report the summary associations of all variants that have reached a certain *P*-value threshold, and recently the release of association estimates in published GWA studies for all measured variants has been advocated [Editorial, 2012]. We investigate methods for using these summarized genetic associations with a risk factor and an outcome to estimate the causal effect of the risk factor on the outcome.

For the causal effect to be consistently estimated, each variant used in a Mendelian randomization analysis must satisfy three assumptions [Didelez and Sheehan, 2007]:

- it is associated with the risk factor,
- it is not associated with any confounder of the risk factor–outcome association,
- it is conditionally independent of the outcome given the risk factor and confounders.

A variant satisfying these assumptions is known as an instrumental variable (IV) [Greenland, 2000]. With a single genetic variant used as an IV and a continuous outcome, assuming all associations are linear, the causal effect of the risk factor on the outcome can be estimated as the ratio of the change in the outcome per additional variant allele divided by the change in the risk factor per additional variant allele [Thomas and Conti, 2004]. With individual-level data, each of these changes can be estimated using linear regression. For a binary outcome, such as disease, a log-linear or other appropriate regression model can be used in the regression of the outcome on the variant [Didelez et al., 2010]. If summarized (aggregated) data are available in the form of these regression coefficients, the ratio estimate of the causal effect can be calculated without recourse to individual-level data [Harbord et al., 2013]. However, with multiple variants, it is not clear how to integrate these genetic association estimates together into a single estimate of the causal effect.

## Methods

We assume that summarized data are available for multiple genetic variants that are single nucleotide polymorphisms (SNPs) and satisfy the IV assumptions for the risk factor of interest *X* and the outcome *Y*. Genetic variant *k*, 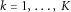 is associated with an observed 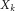 mean change in the risk factor per additional variant allele with standard error 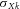 and an observed 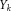 mean change in the outcome per allele with standard error 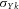 (if the outcome is binary, 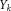 could represent the per allele change in the log-odds or the log-probability of an outcome). Two methods are presented for the estimation of a causal effect using summarized data. The first, which has been previously used in applied investigations, combines the ratio estimates from the individual variants employing inverse-variance weights [Ehret et al., 2011]. The second is a novel likelihood-based method, with independent likelihood contributions from each of the variants.

### Inverse-Variance Weighted Combination of Ratio Estimates

The ratio estimate of the causal effect of *X* on *Y* using genetic variant *k* is 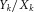. The standard error of the ratio estimate can be approximated using the delta method; the leading term is 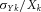 [Thomas et al., 2007]. By using this expression for the standard error, an inverse-variance weighted (IVW) estimate of the causal effect combines the ratio estimates using each variant in a fixed-effect meta-analysis model:

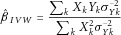
1The approximate standard error of the estimate is:

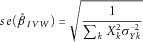
2Further terms from the delta method could be used to improve the estimate of the standard error of the ratio estimates. However, if the estimate of the genetic association with the risk factor is considerably more precise than the estimate of the association with the outcome, as is often the case in practice, then the leading term will dominate.

### Likelihood-Based Method

Alternatively, a model can be constructed by assuming a linear relationship between the risk factor and outcome and a bivariate normal distribution for the genetic association estimates:

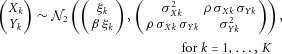
3The causal effect of *X* on *Y*, β, which is assumed to be the same for all genetic variants *k*, can be estimated by direct maximization of the likelihood or by Bayesian methods [Thompson et al., 2005]. The correlation parameter ρ, representing the correlation between genetic association estimates 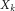 and 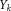 obtained from a single source can be specified as the observational correlation between the risk factor and the outcome. If the estimates 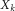 and 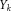 are derived from independent sources, this correlation will be zero. If 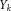 is the per allele change in the log-odds or the log-probability of an outcome, then β represents a log odds ratio or a log relative risk parameter, respectively.

### Independence of Information on Causal Effect From Multiple Variants

By combining the estimates of association from multiple variants into a single estimate of the causal effect, an assumption is made that the variants provide independent information. There are several reasons why this may not be the case. First, the causal estimates are derived from the same data, and so will not be entirely independent. However, correlation between the estimates should be low unless the sample size is particularly small. Secondly, there may be statistical interactions between variants in their associations with the risk factor (gene–gene interactions). Thirdly, the distribution of genetic variants may be correlated (linkage disequilibrium).

We perform simulation studies to assess the impact of the assumption that multiple genetic variants provide independent information on the causal estimate, in particular in the presence of gene–gene interactions and linkage disequilibrium. We compare the causal effect estimate (and its precision) obtained from summarized data to that obtained if individual-level data on the variants, risk factor and outcome were available on the whole study population.

### Simulation Study With Independently Distributed Variants

We initially assume that the genetic variants used as IVs are independently distributed. Individual data were generated for 5,000 participants, indexed by *i*, according to the following model:

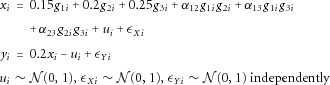
4where the α_.._ parameters represent gene–gene interactions. The causal effect of *X* on *Y* is 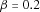, and *U* represents negative (unmeasured) confounding between *X* and *Y*. The IVs 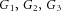 take values 0, 1, 2 representing the number of minor alleles in three independently distributed SNPs, each with a minor allele frequency of 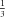. Nine sets of values were taken for the gene–gene interactions: 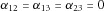 (no gene–gene interactions), and 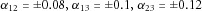 (gene–gene interactions present).

### Simulation Study With Correlated Variants

We repeated the simulation in the absence of gene–gene interactions, but using correlated genetic variants. In order to simulate data on the variants, two random draws 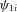 and 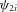 were made from a zero-mean *K*-dimensional multivariate normal distribution for each individual *i*. For each draw, if the *k*th component was positive, a variant allele was recorded for the *k*th variant. The draws represent the two haplotypes for each individual [Lunn et al., 2006]. In this way, the variance-covariance matrix in the multivariate normal distribution determines the correlation between variants 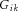, which take values 0, 1, 2. Data for three genetic variants were simulated using the following model:

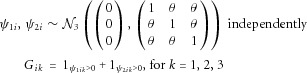
5where 1_._ is an indicator function. We took five values of the correlation parameter 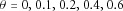 corresponding to a mean squared correlation between variants (*r*^2^) of 0 (no linkage disequilibrium), 0.06, 0.13, 0.26 and 0.41.

### Weak Instruments

A weak instrument is a variable that satisfies the IV assumptions, but does not explain a large proportion of variation in the risk factor, so that the statistical association between the risk factor and the IV in the dataset is ‘weak’ [Burgess et al., 2011b]. IV estimates using weak instruments are biased in the direction of the observational estimate, and the distribution of the IV estimate is poorly approximated by a normal distribution [Burgess and Thompson, 2011]. The magnitude of bias depends on the expected value of the F statistic in the regression of the risk factor on the IVs, with lower F statistics corresponding to greater bias. Bias with a single IV in moderately large datasets is typically negligible, but bias may be considerable when there are multiple IVs [Angrist and Pischke, 2009]. For this reason, we are especially interested in how the summarized data methods perform with weak instruments.

### Implementation

Estimates of the causal effect using all the genetic variants are calculated using individual-level data with the two-stage least squares (2SLS) method [Baum et al., 2003], and using summarized data with the IVW (equations 1 and 2) and likelihood-based (equation 3) methods. The first-stage model in the 2SLS was taken as additive in the variants throughout, and as such the genetic model was misspecified when there were gene–gene interactions. Summarized associations were obtained by ordinary least squares (OLS) linear regression of the risk factor and outcome on each variant in separate regression models. The likelihood-based analyses were performed in R (http://www.r-project.org) using the *optim* command to directly maximize the likelihood.

An estimate of the correlation between the genetic associations with risk factor and outcome of 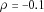 was used based on the approximate observational correlation between the risk factor and outcome. Estimates were not especially sensitive to moderate (±0.2) changes in this correlation. (A sensitivity analysis for this parameter is shown later for an applied example.)

In each scenario, results from 10,000 simulated datasets for the comparison of the individual-level and summarized data methods are given. We present the mean and median estimates across simulations, the standard deviation (SD) of estimates, the mean standard error (SE), the coverage of the 95% confidence interval for the causal effect (the proportion of simulated datasets for which the 95% confidence interval included the true value of 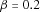), and the empirical power at a 5% significance level (the proportion of simulated datasets for which the 95% confidence interval excluded the null value of 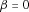). The Monte Carlo standard error (representing the variation in estimates due to the finite number of simulations) was approximately 0.001 for the mean estimate (0.004 for the final scenario with gene–gene interactions) and 0.2% for the coverage. In each set of simulations, the mean value of the F statistic in the regression of the risk factor on the IVs is given.

## Results

### Independently Distributed Variants

Results from the scenario with gene–gene interactions are given in Table1. The individual-level 2SLS and summarized IVW analyses gave similar mean and median estimates, which did not differ in the third decimal place. They showed slight bias in the direction of the observational estimate, consistent with that predicted by weak instrument bias. The likelihood-based analyses showed less bias with mean estimates around or slightly above the true value of 0.2 and median estimates slightly below the true value. Departures from the true value were most marked in the final scenario, where the mean F statistic for the genetic variants is below the conventional threshold of 10, below which IVs are considered to be ‘weak’.

**Table 1:** Results from simulation study with independently distributed variants

α_12_	α_13_	α_23_	Mean F	Method	Mean	Median	SD	Mean SE	Coverage	Power
0	0	0	47.3	2SLS	0.196	0.192	0.085	0.085	94.8	65.2
				IVW	0.196	0.192	0.085	0.078	92.6	70.1
				Likelihood	0.200	0.197	0.087	0.082	94.2	69.1
+0.08	+0.1	+0.12	126.9	2SLS	0.199	0.197	0.052	0.051	95.0	98.3
				IVW	0.199	0.197	0.052	0.047	92.6	98.6
				Likelihood	0.200	0.199	0.052	0.050	94.0	98.6
−0.08	+0.1	+0.12	88.1	2SLS	0.198	0.197	0.061	0.062	95.1	78.1
				IVW	0.198	0.197	0.061	0.057	93.0	81.6
				Likelihood	0.201	0.199	0.062	0.060	94.2	80.9
+0.08	−0.1	+0.12	74.4	2SLS	0.198	0.196	0.068	0.067	95.0	86.6
				IVW	0.198	0.196	0.068	0.062	92.9	88.9
				Likelihood	0.201	0.199	0.068	0.065	94.2	88.6
+0.08	+0.1	−0.12	59.6	2SLS	0.197	0.194	0.075	0.075	94.8	92.4
				IVW	0.197	0.194	0.075	0.069	92.8	93.6
				Likelihood	0.201	0.198	0.076	0.073	94.2	93.4
−0.08	−0.1	+0.12	45.8	2SLS	0.197	0.193	0.085	0.086	95.3	28.9
				IVW	0.197	0.193	0.085	0.079	93.3	37.9
				Likelihood	0.202	0.197	0.087	0.084	94.6	35.9
−0.08	+0.1	−0.12	33.1	2SLS	0.196	0.191	0.102	0.102	94.9	46.7
				IVW	0.196	0.191	0.102	0.093	92.7	53.8
				Likelihood	0.203	0.197	0.105	0.100	94.4	52.2
+0.08	−0.1	−0.12	23.0	2SLS	0.190	0.183	0.123	0.124	94.7	64.3
				IVW	0.190	0.183	0.123	0.113	92.8	69.7
				Likelihood	0.201	0.192	0.129	0.121	94.2	68.7
−0.08	−0.1	−0.12	6.6	2SLS	0.172	0.148	0.249	0.244	93.4	2.8
				IVW	0.172	0.148	0.249	0.217	92.1	10.9
				Likelihood	0.221	0.180	0.357	0.285	94.6	5.7

Instrumental variable estimates of causal effect +0.2 from simulated data with and without gene–gene interactions using individual-level data (two-stage least squares method, 2SLS) and summarized data (inverse-variance weighted, IVW, and likelihood-based methods) with mean F statistic, mean and median estimates across 10,000 simulations, SD of estimates, mean SE of estimates, coverage (%) of 95% confidence interval, and power (%) at a 5% significance level

The coverage was around 95% for the 2SLS and likelihood-based methods, although coverage was slightly underestimated by the 2SLS method in the weak instrument scenario, and was marginally underestimated (average of 94.3%) by the likelihood-based method throughout. Coverage for the IVW method was consistently underestimated at around 93%, indicating that the method gave estimates that were slightly too precise (the mean SE was less than the SD of the estimates). Estimates from the likelihood-based and 2SLS methods had similar efficiency, with the 2SLS analyses giving slightly less variable estimates (lower SD), but the likelihood-based analyses giving slightly more precise estimates (lower mean standard error). The IVW method had the greatest empirical power, although this was offset by the coverage levels not achieving nominal levels. Power from the likelihood-based method was marginally lower, and from the 2SLS lower still.

Overall, despite gene–gene interactions leading to misspecification of the genetic model in the 2SLS method and effect modification in the genetic associations in the summarized data methods, the assumption of independence of the information provided by uncorrelated variants did not seem to give misleading results.

### Correlated Variants

Results from the scenarios with variants in linkage disequilibrium are given in Table2. Estimates from the individual-level and summarized data methods were close to unbiased. The coverage of estimates from the 2SLS method was close to the nominal 95% level; however, the standard errors from the summarized data methods were too small and coverage was well below 95% when variants were correlated, even when the correlation was not large. Power was not reported in this case as it is misleading when the coverage is not close to the nominal levels. This shows that variants used in a summarized analysis must be uncorrelated in order to obtain valid statistical inferences.

**Table 2:** Results from simulation study with correlated variants

*r*^2^	Mean F	Method	Mean	Median	SD	Mean SE	Coverage
0.00	42.6	2SLS	0.195	0.191	0.090	0.090	94.8
		IVW	0.195	0.191	0.090	0.082	92.8
		Likelihood	0.200	0.196	0.092	0.087	94.1
0.06	47.8	2SLS	0.196	0.193	0.086	0.085	94.5
		IVW	0.197	0.194	0.086	0.073	90.3
		Likelihood	0.201	0.198	0.087	0.077	92.0
0.13	52.6	2SLS	0.197	0.194	0.080	0.081	95.0
		IVW	0.199	0.196	0.080	0.066	89.5
		Likelihood	0.202	0.199	0.081	0.070	91.2
0.26	63.3	2SLS	0.197	0.193	0.074	0.073	94.6
		IVW	0.199	0.196	0.074	0.055	85.1
		Likelihood	0.201	0.198	0.074	0.058	87.1
0.41	74.8	2SLS	0.198	0.196	0.067	0.067	95.0
		IVW	0.201	0.199	0.068	0.046	82.1
		Likelihood	0.202	0.200	0.068	0.048	84.2

Instrumental variable estimates of causal effect +0.2 from simulated data with correlated variants (correlation measured by *r*^2^, the average squared correlation between variants) using individual-level data (two-stage least squares method, 2SLS) and summarized data (inverse-variance weighted, IVW, and likelihood-based methods) with mean F statistic, mean and median estimates across simulations, SD of estimates, mean SE of estimates, coverage (%) of 95% confidence interval

### Weak Instruments

We repeated the simulation in the absence of gene–gene interactions and linkage disequilibrium, but using 20 genetic variants with smaller effects on the risk factor to investigate the performance of the methods with weak instruments. Our simulations (see Supporting Information) suggest that the IVW and likelihood-based methods have similar behavior with weak instruments to the 2SLS method. In particular, this means that when the expected value of the F statistic is greater than 10, the bias of the causal estimate is less than 10% of the bias of the confounded observational estimate from an OLS regression analysis [Staiger and Stock, 1997].

## Example: Causal Effect of Low-Density Lipoprotein Cholesterol on Coronary Artery Disease

**Figure 1:**
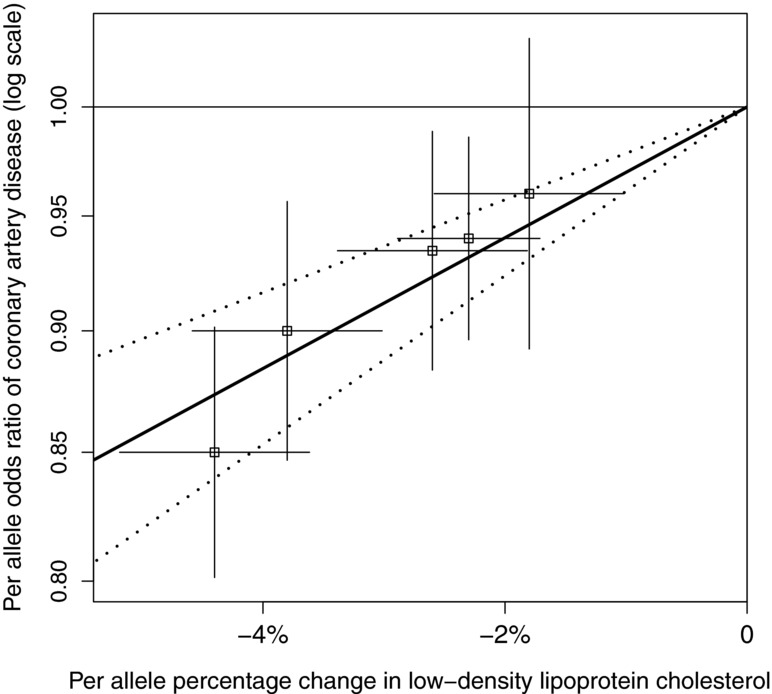
Per allele associations of five genetic variants with low-density lipoprotein cholesterol (LDL-C) and risk of coronary artery disease (CAD) taken from Waterworth et al. [Waterworth et al., 2010], with causal estimate (and 95% confidence interval) of effect of LDL-C on CAD risk (likelihood-based method assuming zero correlation).

Low-density lipoprotein cholesterol (LDL-C) is a known causal risk factor for coronary artery disease (CAD). Genetic variants associated with LDL-C have been found in many different regions of the human genome. We take a published study reporting genetic associations of variants with LDL-C, high-density lipoprotein cholesterol (HDL-C), and triglycerides (TG), and with CAD risk from a meta-analysis of GWA studies [Waterworth et al., 2010]. We consider five genetic variants associated with LDL-C (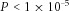), but not associated with HDL-C nor TG (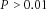) to mitigate against potential pleiotropy. These variants are on chromosome 1 (*PCSK9* and *SORT1* gene regions), chromosome 2 (*APOB*), chromosome 5 (*HMGCR*), and chromosome 19 (*LDLR*); details of the variants are given in the Supporting Information. The variants are not in linkage disequilibrium. A *P*-value of 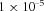 corresponds to an F statistic of around 20, so weak instrument bias is negligible. The estimated associations with 95% confidence intervals are shown graphically in Figure1, together with an estimate of the causal effect of LDL-C on CAD risk using the likelihood-based method for summarized data assuming no correlation between the estimates of genetic association with the risk factor and the outcome. Odds ratio estimates for a 30% reduction in LDL-C levels using the IVW method and the likelihood-based method for a range of different correlation values are displayed in Table3.

**Table 3:** Causal odds ratios of CAD per 30% reduction in LDL-C

Method	Correlation (ρ)	Estimate	95% confidence interval
IVW	–	0.33	0.25, 0.45
Likelihood-based	0	0.33	0.24, 0.46
Likelihood-based	−0.4	0.33	0.23, 0.48
Likelihood-based	−0.2	0.33	0.24, 0.47
Likelihood-based	−0.1	0.33	0.24, 0.46
Likelihood-based	0.1	0.33	0.25, 0.45
Likelihood-based	0.2	0.33	0.25, 0.45
Likelihood-based	0.4	0.34	0.26, 0.44

Instrumental variable estimates of causal effect of low-density lipoprotein cholesterol (LDL-C) on risk of coronary artery disease (CAD) using inverse-variance weighted (IVW) method and likelihood-based method for different values of the correlation parameter (ρ)

We see that the estimates from both summarized data methods are similar, and that changing the correlation parameter in the likelihood-based method has little impact. The graph indicates that variants with a greater magnitude of association with LDL-C also have a greater association with CAD risk. The overall estimate of causal association passes close to the estimate from each of the variants, giving plausibility to the instrumental variable assumptions, and suggesting that changes in LDL-C from different biological mechanisms may have similar effects on CAD risk [Burgess et al., 2012]. The confidence interval from the IVW method was slightly narrower than that from the likelihood-based method with 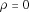, consistent with the slightly reduced coverage seen in the simulation studies.

## Discussion

In this paper, we have considered methods for Mendelian randomization using summarized data on multiple genetic variants. The target for estimation is a non-genetic parameter, the causal effect of the risk factor on the outcome. Each variant provides additional information on this parameter, and so the most precise estimate can be obtained using all available variants that are valid instrumental variables [Burgess et al., 2011aa]. GWA studies are promising resources for powerful Mendelian randomization investigations; however obtaining individual-level data on large numbers of participants is often problematic for reasons of logistics and confidentiality. Our methods for obtaining causal estimates from summarized data commonly presented in published reports facilitate causal assessment of risk factors in existing consortia without additional data collection or sharing. Simulation results suggest that causal estimates obtained from summarized data using a likelihood-based model with independently distributed ‘non-weak’ variants are almost as precise as those obtained from individual-level data, with bias close to zero and coverage close to nominal levels. The empirical power of estimates from the likelihood-based method was greater than that from the 2SLS method. An alternative approach, using an inverse-variance weighted method, gives similar point estimates to an individual-level data analysis and slightly improved power over the likelihood-based method, but slightly too narrow confidence intervals.

### Comparison of Summarized Data Methods

The IVW and likelihood-based methods make different assumptions about the distribution of variables. The IVW method uses an asymptotic estimate of the standard error of the causal (ratio) estimate from each variant; this is known to underestimate the true variation in the estimate, especially when the IV is weak [Burgess and Thompson, 2012]. No allowance is made in the method for uncertainty in the genetic association with the risk factor, although this could be incorporated by including additional terms from the delta method. The likelihood-based method assumes a bivariate normal distribution for the genetic associations with the risk factor and outcome. In both summarized data methods, the variances of the association estimates are assumed to be known; this may be why coverage is consistently slightly underestimated. The likelihood-based method is more computationally complex, but allows for correlation between the genetic association estimates with the risk factor and outcome, which is ignored in the IVW method. The likelihood-based method also has a natural extension to a meta-analysis framework using a hierarchical model (Section 5.6), which may better account for heterogeneity between studies than using meta-analysed genetic association estimates directly.

### Weak Instruments

With weak instruments, estimates using both of the summarized data methods demonstrated bias similar to that of the 2SLS method. If the distributions of genetic variants are uncorrelated, then each variant explains independent variation in the risk factor. For variants of equal strength, the expected F statistic in the univariate linear regression of the risk factor on one of the variants is approximately the same as the expected F statistic in the multiple regression on all of the variants. An F statistic of 10 corresponds to a *P*-value of around 0.001; an F statistic of 20 to a *P*-value of around 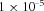; and an F statistic of 30 to a *P*-value of around 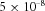, a threshold often used for assessing GWA significance. In the example presented, as the variants were independently distributed and each had a *P*-value of below 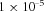, the F statistic for all of the variants is at least 20; in fact, as some variants had *P*-values well below 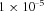, the F statistic would be greater. With a sample size of 10,000, an F statistic of 10 corresponds to a coefficient of determination (*R*^2^) for each variant of around 0.1%. When sample sizes and the strength of genetic variants are limited, it may be necessary to restrict the number of variants used in a Mendelian randomization analysis in order to mitigate against bias from weak instruments.

Close to unbiased estimates with weak instruments can be obtained from individual-level data using the limited information maximum likelihood (LIML) method [Angrist and Pischke, 2009] (see Supporting Information).

### Assessment of the IV Assumptions

A limitation of the use of Mendelian randomization is that the instrumental variable assumptions cannot be assessed without supplementary data. Although the IV assumptions can never be fully tested empirically, they can be assessed to some extent, for example by testing the association of the genetic variants with measured covariates to assess potential pleiotropy. This can be undertaken using individual-level data. It is also possible with summarized data, as with HDL-C and TG in the example, although genetic associations with a full range of covariates would not necessarily be measured or routinely reported by a GWA study. These associations could be checked in the literature; however, the assumption is necessary that the literature-based estimates are valid for the population under investigation. Other assessments, such as addressing population stratification by the evaluation of genetic principal components, or testing for the attenuation of genetic associations with the outcome on adjustment for the risk factor [Glymour et al., 2012], require individual-level data.

In addition, many of the parametric assumptions required by IV methods for effect estimation, such as linearity of genetic associations or of the risk factor–outcome association [Didelez and Sheehan, 2007], cannot be assessed in summarized data.

### Risk Factor and Outcome Associations in Separate Samples

Not all GWA studies measure data on a large number of phenotypic variables, and so genetic associations with the risk factor and the outcome may not be available in the same sample. Estimates of the association of the genetic variants with the risk factor and the outcome may be obtained from independent sources. This is known as a two-sample IV analysis [Pierce and Burgess, 2013]. A key assumption in this case is that the genetic associations with the risk factor and outcome are of the same magnitude in both sources.

Correlation between genetic associations with the risk factor and outcome arising from estimation of the coefficients in the same source is ignored by the inverse-variance weighted method, but can be acknowledged in the likelihood-based method. If the sources for the estimates are neither identical nor disjoint, but instead overlap, then a meta-analysis approach would be recommended to correctly account for the structure in the data. In the absence of study-specific estimates, we advocate the likelihood-based method with a sensitivity analysis for the correlation parameter.

### Linkage Disequilibrium

If two genetic variants are in complete linkage disequilibrium, then inclusion of both variants in an individual-level analysis model would not lead to additional information. However, if variants are in partial linkage equilibrium, although the information provided by each variant is not independent, each variant does provide additional information on the causal effect. Summarized data methods using variants in linkage disequilibrium overstate precision.

Typically, GWA studies report the association of a single lead variant in a genetic region. If this variant is not the causal variant, or if there are multiple causal variants in the region, then additional information may be obtained by considering multiple variants per region. Information on such variants could be included correctly in a summarized analysis by considering conditional effects of variants on the risk factor and outcome by adjustment for the lead variant in a regression model [Yang et al., 2012]. We have not developed these methods as suitable data are not routinely reported in applied analyses.

### Extension to Multiple Studies

The likelihood-based model introduced in this paper can be naturally extended into a hierarchical (multilevel) model in order to combine summarized associations from multiple studies. We assume that genetic variant *k* in study 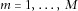 is associated with an observed 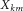 mean change in the risk factor with standard error 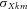, with 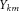 and 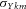 similarly defined for the outcome. Although the presentation of GWA results from multiple studies is common in published research, summarized genetic associations that have been meta-analysed across studies are often reported rather than study-specific associations. We present this model for investigators with access to study-level summarized estimates in the hope that study-specific results will be routinely reported in the future.

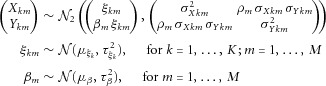
6Random-effects distributions are assumed for the genetic association parameters 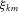 and the causal effect parameters 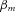; fixed-effect models (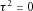) could also be assumed. The overall causal effect of *X* on *Y* is 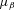. Such models can be estimated in a Bayesian framework with weakly informative priors on the heterogeneity parameters τ^2^. If a particular study does not provide an estimate 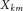, the distribution of the parameter 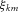 can be estimated using the relevant random-effects distribution as an implicit prior.

### Winner's Curse

The winner's curse is the phenomenon that the association estimate of the variant with the strongest association from a GWA study tends to be overestimated [Göring et al., 2001]. Typically, GWA studies report the single variant from each gene region showing the strongest association with the trait of interest. If several variants have similar strength, the variant with the strongest observed association will not always truly have the strongest association with the risk factor. Data-driven selection of the ‘lead’ variant results in bias in the causal estimate, as the association of the lead variant is typically over-estimated [Burgess et al., 2011b]. This is an example of selection bias. This bias can be eliminated by choosing the lead variant for each genetic region from an independent data source.

### Sample Code

Sample code for estimating causal effects from summarized data for multiple variants associated with a risk factor in a single study and in multiple studies for R and WinBUGS is provided in the Supporting Information.

## Conclusion

If individual-level data are available, these should be used directly to perform a Mendelian randomization analysis. However, if individual-level data are not available, then valid statistical inference can still be obtained from summarized data on the associations of genetic variants with the risk factor and the outcome. On the basis of the simulations in this paper and the theoretical explanations for the differences in results, we recommend the likelihood-based model for applied analysis of summarized data. However, analyses should be restricted to uncorrelated genetic variants (no linkage disequilibrium), and care should be taken when including large numbers of variants to assess potential weak instrument bias by examining the F statistic in the regression of the risk factor on the variants.

## Supporting Information

As a service to our authors and readers, this journal provides supporting information supplied by the authors. Such materials are peer reviewed and may be re-organized for online delivery, but are not copy-edited or typeset. Technical support issues arising from supporting information (other than missing files) should be addressed to the authors.
