# Supplementary material for: Mendelian Randomization Analysis With Multiple Genetic Variants Using Summarized Data
Source: Genet Epidemiol. 2013 Sep 20;37(7):658–65. doi: 10.1002/gepi.21758 (PMC4377079; doi:10.1002/gepi.21758)
Supplement: Supplementary file 1 — supplementary material [file gepi0037-0658-sd1.zip › networkthirdfigures.pdf]

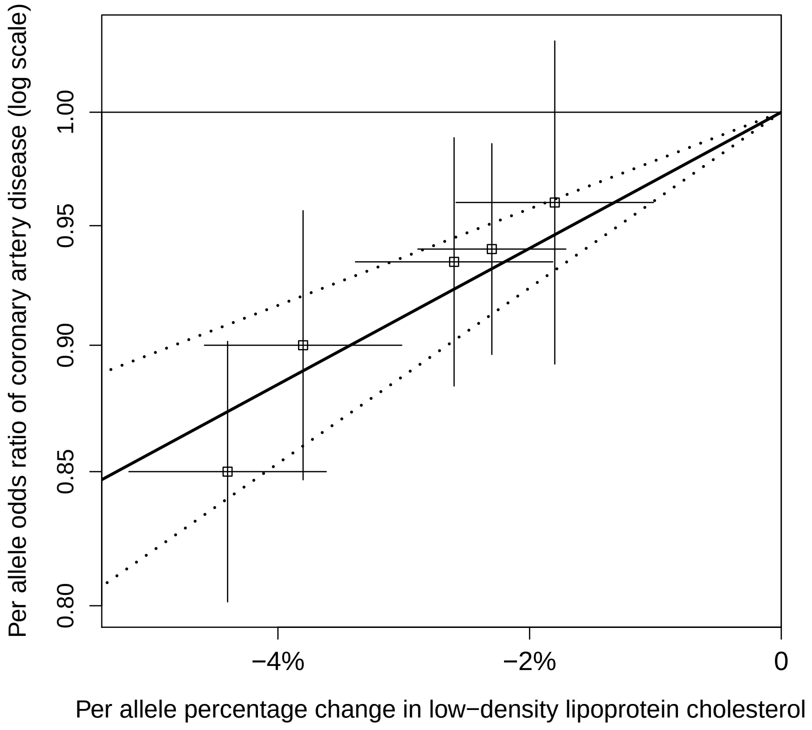

Figure 1: (see end of manuscript)

Figure legends

Figure 1: Per allele associations of five genetic variants with low-density lipoprotein cholesterol (LDL-C) and risk of coronary artery disease (CAD) taken from Waterworth et al. [Waterworth et al., 2010], with causal estimate (and 95% confidence interval) of effect of LDL-C on CAD risk (likelihood-based method assuming zero correlation).

Reference

Waterworth, D., Ricketts, S., Song, K., Chen, L., Zhao, J., Ripatti, S., Aulchenko, Y., Zhang, W., Yuan, X., Lim, N., et al. 2010. Genetic variants influencing circulating lipid levels and risk of coronary artery disease. *Arteriosclerosis, Thrombosis, and Vascular Biology*, 30(11):2264–2276.
