# Supplementary material for: Mendelian Randomization Analysis With Multiple Genetic Variants Using Summarized Data
Source: Genet Epidemiol. 2013 Sep 20;37(7):658–65. doi: 10.1002/gepi.21758 (PMC4377079; doi:10.1002/gepi.21758)
Supplement: Supplementary file 1 — supplementary material [file gepi0037-0658-sd1.zip › networkthirdtables2.pdf]

Table I: **Results from simulation study with independently distributed variants**

α_{12}	α_{13}	α_{23}	Mean F	Method	Mean	Median	SD	Mean SE	Coverage	Power
				2SLS	0.196	0.192	0.085	0.085	94.8	65.2
0	0	0	47.3	IVW	0.196	0.192	0.085	0.078	92.6	70.1
				Likelihood	0.200	0.197	0.087	0.082	94.2	69.1
				2SLS	0.199	0.197	0.052	0.051	95.0	98.3
+0.08	+0.1	+0.12	126.9	IVW	0.199	0.197	0.052	0.047	92.6	98.6
				Likelihood	0.200	0.199	0.052	0.050	94.0	98.6
				2SLS	0.198	0.197	0.061	0.062	95.1	78.1
−0.08	+0.1	+0.12	88.1	IVW	0.198	0.197	0.061	0.057	93.0	81.6
				Likelihood	0.201	0.199	0.062	0.060	94.2	80.9
				2SLS	0.198	0.196	0.068	0.067	95.0	86.6
+0.08	−0.1	+0.12	74.4	IVW	0.198	0.196	0.068	0.062	92.9	88.9
				Likelihood	0.201	0.199	0.068	0.065	94.2	88.6
				2SLS	0.197	0.194	0.075	0.075	94.8	92.4
+0.08	+0.1	−0.12	59.6	IVW	0.197	0.194	0.075	0.069	92.8	93.6
				Likelihood	0.201	0.198	0.076	0.073	94.2	93.4
				2SLS	0.197	0.193	0.085	0.086	95.3	28.9
−0.08	−0.1	+0.12	45.8	IVW	0.197	0.193	0.085	0.079	93.3	37.9
				Likelihood	0.202	0.197	0.087	0.084	94.6	35.9
				2SLS	0.196	0.191	0.102	0.102	94.9	46.7
−0.08	+0.1	−0.12	33.1	IVW	0.196	0.191	0.102	0.093	92.7	53.8
				Likelihood	0.203	0.197	0.105	0.100	94.4	52.2
				2SLS	0.190	0.183	0.123	0.124	94.7	64.3
+0.08	−0.1	−0.12	23.0	IVW	0.190	0.183	0.123	0.113	92.8	69.7
				Likelihood	0.201	0.192	0.129	0.121	94.2	68.7
				2SLS	0.172	0.148	0.249	0.244	93.4	2.8
−0.08	−0.1	−0.12	6.6	IVW	0.172	0.148	0.249	0.217	92.1	10.9
				Likelihood	0.221	0.180	0.357	0.285	94.6	5.7

Instrumental variable estimates of causal effect +0.2 from simulated data with and without gene–gene interactions using individual-level data (two-stage least squares method, 2SLS) and summarized data (inverse-variance weighted, IVW, and likelihood-based methods) with mean F statistic, mean and median estimates across 10 000 simulations, standard deviation (SD) of estimates, mean standard error (SE) of estimates, coverage (%) of 95% confidence interval, and power (%) at a 5% significance level

Table II: **Results from simulation study with correlated variants**

r^2	Mean F	Method	Mean	Median	SD	Mean SE	Coverage
0.00	42.6	2SLS	0.195	0.191	0.090	0.090	94.8
		IVW	0.195	0.191	0.090	0.082	92.8
		Likelihood	0.200	0.196	0.092	0.087	94.1
0.06	47.8	2SLS	0.196	0.193	0.086	0.085	94.5
		IVW	0.197	0.194	0.086	0.073	90.3
		Likelihood	0.201	0.198	0.087	0.077	92.0
0.13	52.6	2SLS	0.197	0.194	0.080	0.081	95.0
		IVW	0.199	0.196	0.080	0.066	89.5
		Likelihood	0.202	0.199	0.081	0.070	91.2
0.26	63.3	2SLS	0.197	0.193	0.074	0.073	94.6
		IVW	0.199	0.196	0.074	0.055	85.1
		Likelihood	0.201	0.198	0.074	0.058	87.1
0.41	74.8	2SLS	0.198	0.196	0.067	0.067	95.0
		IVW	0.201	0.199	0.068	0.046	82.1
		Likelihood	0.202	0.200	0.068	0.048	84.2

Instrumental variable estimates of causal effect +0.2 from simulated data with correlated variants (correlation measured by r^2, the average squared correlation between variants) using individual-level data (two-stage least squares method, 2SLS) and summarized data (inverse-variance weighted, IVW, and likelihood-based methods) with mean F statistic, mean and median estimates across simulations, standard deviation (SD) of estimates, mean standard error (SE) of estimates, coverage (%) of 95% confidence interval

Table III: **Causal odds ratios of CAD per 30% reduction in LDL-C**

Method	Correlation (ρ)	Estimate	95% confidence interval
IVW	-	0.33	0.25, 0.45
Likelihood-based	0	0.33	0.24, 0.46
Likelihood-based	-0.4	0.33	0.23, 0.48
Likelihood-based	-0.2	0.33	0.24, 0.47
Likelihood-based	-0.1	0.33	0.24, 0.46
Likelihood-based	0.1	0.33	0.25, 0.45
Likelihood-based	0.2	0.33	0.25, 0.45
Likelihood-based	0.4	0.34	0.26, 0.44

Instrumental variable estimates of causal effect of low-density lipoprotein cholesterol (LDL-C) on risk of coronary artery disease (CAD) using inverse-variance weighted (IVW) method and likelihood-based method for different values of the correlation parameter (ρ)
