# Supplementary material for: Mendelian Randomization Analysis With Multiple Genetic Variants Using Summarized Data
Source: Genet Epidemiol. 2013 Sep 20;37(7):658–65. doi: 10.1002/gepi.21758 (PMC4377079; doi:10.1002/gepi.21758)
Supplement: Supplementary file 1 — supplementary material [file gepi0037-0658-sd1.zip › networkthirdappendix.pdf]

Web Appendix

A.1 Simulation study with weak instruments

We repeated the simulation of Section 2.4 in the absence of gene–gene interactions and linkage disequilibrium, but using 20 genetic variants with smaller effects on the risk factor to investigate the performance of the methods with weak instruments. The associations of the variants α_k with the risk factor were drawn from a normal distribution with mean 0.07 and variance 0.02^2. These values were chosen so that the average proportion of variation in the risk factor explained by the variants (the coefficient of determination, R^2) was approximately the same as in the initial simulation without gene–gene interactions (2.7%). We took two values of the causal effect ($\beta = 0, 0.2$), additionally considering a null causal association, and varied the direction of the unmeasured confounding ($\beta_U = \pm 1$) to investigate the potential direction of bias from weak instruments.

$$
\begin{aligned}
x_i &= \sum_{k=1}^{20} \alpha_k g_{ik} + u_i + \epsilon_{Xi} \qquad (1) \\
\alpha_k &\sim \mathcal{N}(0.07, 0.02^2) \text{ independently} \\
y_i &= \beta x_i + \beta_U u_i + \epsilon_{Yi}
\end{aligned}
$$

In the weak instruments scenario, maximization of the likelihood was computationally expensive, as there were 21 parameters to optimize over, and so the likelihood-based analyses were performed in WinBUGS (http://www.mrc-bsu.cam.ac.uk/bugs/) in a Bayesian setting. The parameter ρ was taken as the observational correlation in the dataset under analysis. In addition to the individual-data two-stage least squares (2SLS) method, the limited information maximum likelihood (LIML) method was performed, as this is known to give close to unbiased estimates with weak instruments [Baum et al., 2003; Angrist and Pischke, 2009]. Although the theoretical mean LIML estimate is undefined as it tends to infinity, we did not see any extreme values of

the estimator in the simulations considered, and so the mean LIML estimates across simulations are reported.

In the Bayesian analyses, 'vague' prior distributions were used: normal distributions with mean zero and variance 1000^2 for the estimated parameters. The mean of the posterior distribution was regarded as the 'estimate', the standard deviation of the posterior distribution as the 'standard error (SE)', and the 2.5th to the 97.5th percentile range as the '95% confidence interval'.

Results are given in Web Table A1. Although the 2SLS and likelihood-based summarized data method seem to give correctly-sized confidence intervals, with the standard deviation and mean standard error of estimates almost equal, the coverage is poor due to bias in the parameter estimate. When the coverage is not close to the nominal value of 95%, power is a misleading criterion to judge between methods, as the confidence intervals are known to be underestimated. In the IVW method, bias is similar to that in the 2SLS method, and the standard deviation and mean standard error of estimates differ. In contrast, the LIML method gives median unbiased estimates. Confidence intervals from the LIML method are slightly too narrow, although coverage is close to 95% (average of 93.8%) throughout.

A.2 R code for combining summarized data on multiple variants

We provide R code for combining estimates of genetic association with a risk factor and with an outcome into a causal estimate of the effect of the risk factor on the outcome. For a valid causal estimate, it is required that the genetic variants are true instrumental variables for the risk factor. The code provided is for three genetic variants. Variant 1 has association `ax1` (standard error `ax1se`) with the risk factor, and `ay1` (standard error `ay1se`) with the outcome; variables are defined similarly for variants 2 and 3. The correlation between the risk factor and outcome associations is `rho`.

Web Table A1: **Results from simulation study with weak instruments**

β	β_U	Mean F	Method	Mean	Median	SD	Mean SE	Coverage	Power
0	+1	6.9	2SLS	0.068	0.069	0.084	0.084	84.3	15.7
			LIML	-0.005	-0.001	0.102	0.094	93.7	6.3
			IVW	0.067	0.069	0.084	0.086	86.8	13.2
			Likelihood	0.060	0.062	0.083	0.083	87.5	12.5
0	−1	6.9	2SLS	-0.066	-0.068	0.083	0.084	85.2	14.8
			LIML	0.007	0.003	0.101	0.094	93.6	6.4
			IVW	-0.065	-0.067	0.083	0.086	87.8	12.2
			Likelihood	-0.059	-0.061	0.082	0.083	88.4	11.6
+0.2	+1	6.9	2SLS	0.268	0.270	0.084	0.084	84.4	86.1
			LIML	0.195	0.200	0.101	0.094	94.0	56.6
			IVW	0.267	0.270	0.084	0.096	91.4	81.7
			Likelihood	0.261	0.263	0.082	0.083	87.7	83.1
+0.2	−1	6.9	2SLS	0.134	0.131	0.084	0.084	84.3	34.0
			LIML	0.207	0.202	0.102	0.094	94.0	60.5
			IVW	0.134	0.131	0.084	0.079	82.9	39.7
			Likelihood	0.141	0.139	0.083	0.083	87.9	42.4

Instrumental variable estimates of causal effect $\beta = 0$ or $\beta = 0.2$ from simulated data with weak instruments varying the direction of confounding β_U using individual-level data (two-stage least squares, 2SLS, and limited information maximum likelihood, LIML, methods) and summarized data (inverse-variance weighted, IVW, and likelihood-based methods) with mean F statistic, mean and median estimates across simulations, standard deviation (SD) of estimates, mean standard error (SE) of estimates, coverage (%) of 95% confidence interval, and power (%) at a 5% significance level

```
likelihood <- function(pr) {
 Sigma1 = matrix(c(ax1se^2, rho*ax1se*ay1se, rho*ax1se*ay1se, ay1se^2),
                  nrow=2, ncol=2)
 Sigma2 = matrix(c(ax2se^2, rho*ax2se*ay2se, rho*ax2se*ay2se, ay2se^2),
                  nrow=2, ncol=2)
 Sigma3 = matrix(c(ax3se^2, rho*ax3se*ay3se, rho*ax3se*ay3se, ay3se^2),
                  nrow=2, ncol=2)
 return(1/2*log(abs(det(Sigma1)))+1/2*t(c(ax1-pr[1], ay1-pr[4]*pr[1]))%*%
          solve(Sigma1)%*%c(ax1-pr[1], ay1-pr[4]*pr[1])+
       1/2*log(abs(det(Sigma2)))+1/2*t(c(ax2-pr[2], ay2-pr[4]*pr[2]))%*%
          solve(Sigma2)%*%c(ax2-pr[2], ay2-pr[4]*pr[2])+
       1/2*log(abs(det(Sigma3)))+1/2*t(c(ax3-pr[3], ay3-pr[4]*pr[3]))%*%
          solve(Sigma3)%*%c(ax3-pr[3], ay3-pr[4]*pr[3])) }
opt1 = optim(c(0,0,0,0), likelihood, hessian=TRUE)
opt1$par[4]                    # point estimate
sqrt(solve(opt1$hessian)[4,4]) # standard error
```

A.3 WinBUGS code for combining summarized data on multiple variants in a single study

We provide WinBUGS code for combining estimates of genetic association with a risk factor and with an outcome into a causal estimate of the effect of the risk factor on the outcome. For a valid causal estimate, it is required that the genetic variants are true instrumental variables for the risk factor. The code provided is for K genetic variants. The association of genetic variant k with the risk factor is `x[k,1]` and the association with the outcome is `x[k,2]`, and standard errors are `sigma[k,1:2]`. The correlation between the risk factor and outcome associations is `rho`.

```
model {
 beta1 ~ dnorm(0, 0.000001)
```

```
 for (k in 1:K) {
  Sigma0[k,1,1] <- pow(sigma[k,1], 2)
  Sigma0[k,1,2] <- sigma[k,1]*sigma[k,2]*rho
  Sigma0[k,2,1] <- sigma[k,1]*sigma[k,2]*rho
  Sigma0[k,2,2] <- pow(sigma[k,2], 2)
  Tau0[k,1:2,1:2] <- inverse(Sigma0[k,1:2,1:2])

  x[k,1:2] ~ dmnorm(xi[k,1:2], Tau0[k,1:2,1:2])
  xi[k,1] ~ dnorm(0, 0.000001)
  xi[k,2] <- beta1*xi[k,1]
  }
}
```

A.4 WinBUGS code for combining summarized data on multiple variants in a meta-analysis

We provide WinBUGS code for combining estimates of genetic association with a risk factor and with an outcome into a causal estimate of the effect of the risk factor on the outcome in a meta-analysis of multiple studies. For a valid causal estimate, it is required that the genetic variants are true instrumental variables for the risk factor. The code provided is for K genetic variants in M studies. The associations of genetic variant k with the risk factor in study m is `x[m,k,1]` and with the outcome is `x[m,k,2]`, and standard errors are `sigma[m,k,1:2]`. The correlation between between the risk factor and outcome associations in study m is `rho[m]`.

```
model {
 mubeta1         ~ dnorm(0, 0.000001)
 sigtaubeta1     ~ dunif(0,1)
 prectaubeta1  <- pow(sigtaubeta1, -2)
```

```
 for (k in 1:K) {
  muxi[k]       ~ dnorm(0, 0.000001)
  sigtauxi[k]   ~ dunif(0,1)
  prectauxi[k] <- pow(sigtauxi[k], -2)
  }

 for (m in 1:M) {
  beta1[m] ~ dnorm(mubeta1, prectaubeta1)

   for (k in 1:K) {
    Sigma0[m,k,1,1] <- pow(sigma[m,k,1], 2)
    Sigma0[m,k,1,2] <- sigma[m,k,1]*sigma[m,k,2]*rho[m]
    Sigma0[m,k,2,1] <- sigma[m,k,1]*sigma[m,k,2]*rho[m]
    Sigma0[m,k,2,2] <- pow(sigma[m,k,2], 2)
    Tau0[m,k,1:2,1:2] <- inverse(Sigma0[m,k,1:2,1:2])

    x[m,k,1:2] ~ dmnorm(xi[m,k,1:2], Tau0[m,k,1:2,1:2])
    xi[m,k,1]  ~ dnorm(muxi[k], prectauxi[k])
    xi[m,k,2]  <- beta1[m]*xi[m,k,1]
    }
  }
}
```

A.5 Data on genetic variants and associations used in applied example

Details of the genetic variants used in the applied example of the main manuscript, and their associations with low-density lipoprotein cholesterol (LDL-C) and risk of

coronary artery disease (CAD) are given in Web Table A2. In the analyses, the log-odds ratio of CAD was assumed to be normally distributed, and its standard error was estimated by dividing the width of the confidence interval on the log scale by 3.92.

Web Table A2: **Genetic variants used in example**

Genetic variant (relevant gene)	Per allele change in log-transformed low-density lipoprotein cholesterol (standard error)	Per allele odds ratio of coronary artery disease (95% confidence interval)
rs11206510 (*PCSK9*)	−0.026 (0.004)	0.93 (0.88, 0.99)
rs660240 (*SORT1*)	−0.044 (0.004)	0.85 (0.80, 0.90)
rs515135 (*APOB*)	−0.038 (0.004)	0.90 (0.85, 0.96)
rs12916 (*HMGCR*)	−0.023 (0.003)	0.94 (0.90, 0.99)
rs2738459 (*LDLR*)	−0.018 (0.004)	0.96 (0.89, 1.03)

Genetic variants used in applied example and their associations with log-transformed low-density lipoprotein cholesterol and coronary artery disease risk taken from Waterworth et al. [Waterworth et al., 2010]

References

Angrist, J. and Pischke, J. 2009. *Mostly harmless econometrics: an empiricist's companion. Chapter 4: Instrumental variables in action: sometimes you get what you need.* Princeton University Press.

Baum, C., Schaffer, M., and Stillman, S. 2003. Instrumental variables and GMM: Estimation and testing. *Stata Journal*, 3(1):1–31.

Waterworth, D., Ricketts, S., Song, K., Chen, L., Zhao, J., Ripatti, S., Aulchenko, Y., Zhang, W., Yuan, X., Lim, N., et al. 2010. Genetic variants influencing circulating

lipid levels and risk of coronary artery disease. *Arteriosclerosis, Thrombosis, and Vascular Biology*, 30(11):2264–2276.
